# Does education finance reduce the inequality of educational results? The mediation effect of shadow education

**DOI:** 10.3389/fpsyg.2022.1041615

**Published:** 2022-10-28

**Authors:** Yingqi Ma, Wei Jia, Jingxuan Wang, Xuesong Wang, Yuanxiang Zhou, Zeran Yan

**Affiliations:** ^1^School of Finance, Anhui University of Finance and Economics, Bengbu, Anhui, China; ^2^College of Politics and Public Administration, Qingdao University, Qingdao, Shandong, China; ^3^Institute of Finance and Public Management, Anhui University of Finance and Economics, Bengbu, Anhui, China; ^4^State Grid Electricity Research Institute, Beijing, China; ^5^Institute of Statistics and Applied Mathematics, Anhui University of Finance and Economics, Bengbu, Anhui, China

**Keywords:** mediation effect model, microheterogeneous, shadow education, educational results equality, public education finance

## Abstract

Public education finance in China plays an important role in education equality. This study investigated two mediation effects with a generalized structural equation model that comprised the mediation effect of shadow education at the school, family, and individual levels and the moderating role of education finance. There was a strong association among heterogeneity factors, shadow education, and educational results, with shadow education playing a mediating role in math and English courses. Individual heterogeneity differences had a negative impact on equality in educational results through access to additional shadow education opportunities, while heterogeneous factors were mediated through shadow education, causing financial moderation effects, in turn affecting inequality in educational results. Finally, the moderation degree and direction of education finance varied significantly, with a greater moderation effect on household-level factors that lead to unequal educational results. Targeted efforts are required to regulate shadow education, which is key to the development of the education system.

## Introduction

Equality in education encompasses three levels: starting point, process, and results. Equality expresses both equal access to and equal treatment in the education system, meaning educational equality is ultimately about ensuring that students from different societal levels have equal access to education, academic achievement, and prospects for future success. This study starts from the unique perspective of shadow education, connoting three aspects of comprehensive education equality to explore the key role played by education finance investment in the equality of educational results. This paper clarifies a path for achieving educational equality through better policy.

According to the 2019 National Statistical Bulletin on Education Development, China’s nine-year compulsory education system comprises 212,600 schools, 154 million students, and 10.0165 million full-time teachers. However, in the process of popularizing compulsory education, problems have arisen such as the uneven development of education that seriously affects education equality and the realization of social equality. Educational inequality stems from many sources, from the micro-level of the heterogeneity of individuals to the macro-level of the distribution of educational resources. The Chinese government has begun to look at educational equality and the unbalanced development of education, but a core problem is how to reduce it and especially how to reduce the inequality of educational results.

From the micro point of view, educational inequality stems from differences in individual endowment, family investment, and school quality (Xiong et al., 2014). Many studies have shown that educational financial investment plays a key role in educational equality (Hanushek and Raymond, 2005; Xue, 2011; Jackson et al., 2015; Lynne et al., 2016; Ma and Jin, 2016; Wang, 2017; Hu, 2018). The key problem is the imbalanced resource distribution between regions, urban and rural areas, and even between schools and individuals, all of which need to be addressed. With the sustainable development of the economy, society’s demand for high-quality human capital is increasing, and families are focusing more on educational investment. Differences in the allocation of educational finance may be the key factor that motivates families to invest in education. In recent years, shadow education has rapidly developed because of the uneven distribution of high-quality educational resources, and together with formal school education, it provides educational resources for individuals and has gradually become the main investment that families make in education (Bray, 2006; Li and Hu, 2017). However, shadow education weakens the social reproduction function of school education and may become the new mediator of intergenerational transmission of education (Xue, 2015). Due to family investment, shadow education is likely to lead to relatively higher returns, with families of higher socio-economic status having greater incentives to invest in it. Against the background of the development of education marketization, this both adds to students’ academic burdens and forms a new mechanism for the generation of education inequality, weakening government policies to promote equal results. This study explores the mediation mechanism of shadow education in the realization of educational outcome equality and ascertains whether national public Education Financial Input can reduce the potential educational outcome inequality resulting from shadow education.

## Literature review

Stevenson and Baker (1992) coined the concept of ‘shadow education’ to describe education that complements and takes place outside of formal schooling to help students achieve higher academic standing. Recent research has shown that imbalances in the allocation of educational resources are important reasons for the formation of the shadow education market.

Studies on shadow education and educational equality have covered various topics. First, some studies have examined how individual background differences relate to shadow education participation. According to Kwak (2004) study in South Korea, the higher the level of public school education, the lower the demand for shadow education. Other studies have also concluded that the degree of economic development (Silova, 2010; Song et al., 2013), quality of school education (Bray and Kwok, 2003; Kim and Lee, 2010), family socio-economic background (Bray and Kwok, 2003; Yamamoto and Brinton, 2010; Xue, 2015), hope and life satisfaction (Ji et al., 2022), and student performance (Choi, 2012) result in different degrees of participation in shadow education.

Second, other factors that influence participation in shadow education have been investigated. Weng (2017) summarized the current research on shadow education and found that supply and demand for shadow education were affected by both macroeconomic and micro factors; the existing literature is mainly aimed at the latter topic, with micro factors explaining the heterogeneity between different families’ shadow education demand. Among the many micro factors, the most critical are economic expenditure, location, age, parents’ educational levels, and educational results, which can be summarized by three levels: individual, school, and family (Dang and Rogers, 2008). Studies of the individual level have shown that students with higher performance are more likely to participate in shadow education (Kim and Lee, 2010; Choi, 2012; Xue, 2015), which extends and reinforces the inequality of school education. At the school level, the focus has been on the quantity and quality of teachers, school types, and the natural experiments that formed by education-related policies (Dang and Rogers, 2008; Kim and Lee, 2010).

The third type of research has focused on the influence of shadow education on educational results. There are different measures of shadow education (whether to participate, for how long, and expenditure thereon), although educational results are generally measured by years of education and individuals’ school performances. Briggs (2001) concluded that market-oriented exam preparation courses had inconsistent effects on the scores of different types of courses but had a positive effect on the improvement of most courses. Similarly, Buchmann (2002) studied Kenyan students and found that shadow education improved their educational results and significantly reduced the possibility of grade retention, while Ryu and Kang (2013) concluded that shadow education had either no influence or a negative influence on educational results. In recent years, Chinese scholars have also carried out corresponding studies (Xue et al., 2014; Hu et al., 2015; Xue, 2018) discussing the heterogeneity of shadow education in the equality of educational results by combining micro factors.

The present study contributes in the following respects. Little literature has taken macro factors into account, particularly the role of national educational financial input into educational results equality (Chen, 2019; Sheng, 2019; Zhou et al., 2019; Zou, 2020; Hu and Cai, 2021). Zhang (2020) based on the data of provincial education expenditure per student in primary and junior high schools from 1995 to 2016, combined the comparative thinking of the event research method and the double difference method, and used the linear relationship between the education expenditure per student in the western and eastern regions to show the western region. Under the influence of the central policy, the region has produced significant excess education expenditure per student. Although Chen and Zhi (2017) discussed whether financial investment in education can effectively reduce the inequality of educational results, it was not from the perspective of shadow education mediation. Unlike formal school education, shadow education is characterized by high costs and uncertain benefits. Based on rational choice theory, families take these two aspects into account in considering family education investment. If shadow education’s features restrict the free decision-making of family education investment, will national education finance play a regulating role? In this study, extensibility research is conducted to explore this issue.

## Materials and methods

We used the CEPS of the National Survey Research Center at Renmin University of China (NSRC) 2013–2014 for our empirical analysis. The CEPS aims to record students’ family, school, and community background and the influence of the macro-social structure on personal education output. The survey mainly sampled grades 7 and 9 (the first and third grades of junior high school) and randomly selected 28 county-level units (counties, districts, and cities) across China.

Based on the existing literature, the present study included heterogeneous factors from the individual, family, and school levels, as follows:

### Explained variables

We took students’ individual test scores as the measurement of educational results (Hu et al., 2015) and simultaneously examined the four key aspects of students’ individual quality cultivation—cognitive ability, Chinese, math, and English scores—to more carefully judge the fairness of educational results.

### Explanatory variables

According to the research purpose and design ideas of this paper, the included explanatory variables can be roughly divided into three categories.

The first category is the national education financial investment at the macro level, and the corresponding questionnaire question is “How much is the school’s average financial allocation for junior high school students this year?”

The second category is the situation of shadow education, which includes “tutoring that only considers academic courses,” “tutoring that only considers interest courses” and “all tutoring.” No measure, and the other two cases are discussed further later.

The third category is the selection of heterogeneity indicators, which includes: (1) Individual level: gender (male = 1), grades (ninth grades = 1), ethnic groups (Han nationality = 1), household registration type (agriculture = 1), only child status (only child = 1), student achievement ranking (ordered score, 1–5), self-education expectations (ordered score, 1–10); (2) Family level: family economic level (difficulty = 1; moderate = 2; wealthy = 3), and the occupational level of the parents (ordered score, 1–3). According to the classification standards of Li and Liu (2020), the occupational level of the individual parents of the survey data is summarized as the basic level (Including general workers in production and manufacturing, general workers in commerce and service industries, farmers, unemployed and laid-off), middle class (including teachers, engineers, doctors/lawyers, skilled workers, self-employed) and dominant class (including leaders of state organs and institutions and staff, middle and senior managers of enterprises/company), parental education level (ranked score, 1–9, represented by the highest educational level of both father and mother), parental political capital (member of the Communist Party of China = 1), parents Educational expectations (ordered score, 1–10), family cultural capital (including “level of book ownership (ordered score, 1–5), computer and Internet ownership [ordered score, 1–5), and “whether parents provide Help (providing help = 1)”]; (3) School level: school ranking (ordered score, 1–3), school type (public school = 1), school size (including class size, number of students, number of teachers, continuous variable). Finally, by deleting some samples containing missing values, we get 12,714 available data samples, which forms a necessary condition for large-sample statistical inference. The specific variable descriptive statistics are shown in Table 1.

**Table 1:** Descriptive statistics.

Variable name	Obs.	Mean	SD	Min	Max
Cognitive score	12,714	0	0.84	−2.03	2.71
Chinese score	12,714	70.53	9.60	9.96	98.47
Math score	12,714	70.44	9.71	25.71	145.11
English score	12,714	70.50	9.74	14.24	107.82
Shadow education	12,714	0.45	0.50	0	1
Education financial input	12,714	958.40	697.27	0	3,850
Gender	12,714	0.50	0.50	0	1
Grade	12,714	0.47	0.50	0	1
Ethnicity	12,714	0.94	0.24	0	1
Hukou	12,714	0.56	0.50	0	1
One child	12,714	0.42	0.49	0	1
Performance ranking	12,714	3.11	1.11	1	5
Self-education expectation	12,714	6.91	1.74	1	10
Family economic level	12,714	1.84	0.50	1	3
Parent class level	12,714	1.71	0.70	1	3
Parent education level	12,714	4.45	2.00	1	9
Parent political capital	12,714	0.11	0.31	0	1
Parent expected education	12,714	6.73	1.67	1	10
Having books	12,714	3.14	1.21	1	5
Having computers and internet	12,714	1.26	0.92	0	2
Parental academic assistance	12,714	0.78	0.41	0	1
School ranking	12,714	2.01	0.64	1	3
School type	12,714	0.94	0.23	0	1
School size	12,714	1059.55	621.03	110	2,925
Class size	12,714	21.21	10.56	3	46
Teacher numbers	12,714	87.72	39.40	15	181

## Results

### Empirical and heterogeneity analysis

This study investigated the relationship among heterogeneous factors, the acquisition of shadow education, and educational results to deduce the effect of different heterogeneous factors on the acquisition of shadow education, and whether this acquisition is independent of the results of school education. Table 2 shows the independent results between the different heterogeneous factors and the acquisition of shadow education. Specifically, girls participate proportionately more than boys, lower grade students are more likely to engage in extra-class tutoring, there are more Han ethnic students participating than minority students, and urban students prefer shadow education more than rural students. Furthermore, the participation rate of one-child families is about 1.76 times that of non-one-child families, and the better-performing students are more likely to participate in shadow education.

**Table 2:** Heterogeneity difference of shadow education.

Levels	Variables	Classification	Proportion of shadow education participation (%)	Chi-square test
Individual level	Gender	Male	43.03	$\chi^{2}=$ 22.76
		Female	47.25	
	Grade	9	42.54	$\chi^{2}=$ 31.42
		7	47.49	
	Ethnicity	Han	45.49	$\chi^{2}=$ 9.62
		Minority	39.85	
	Hukou	Rural	33.53	$\chi^{2}=$ 896.71
		Non-rural	60.19	
	One child	Yes	59.48	$\chi^{2}=$ 757.53
		No	34.85	
	Performance ranking	Bad	36.34	$\chi^{2}=$ 83.86
		Lower	43.11	
		Middle	44.05	
		Upper	47.71	
		Great	53.35	
	Self-education expectation	Low	31.42	$\chi^{2}=$ 49.14
		Middle	28.56	
		High	49.14	
Family level	Family economic status	Difficult	29.78	$\chi^{2}=$ 425.51
		Medium	48.15	
		Affluent	66.29	
	Parent occupation	Low	30.79	$\chi^{2}=$ 1100.32
		Middle	50.07	
		High	73.71	
	Parent education	Low	32.26	$\chi^{2}=$ 1400.11
		Middle	50.50	
		High	76.84	
	Parents’ political status	CCP	62.40	$\chi^{2}=$ 191.62
		No CCP	42.98	
	Parents’ education expectation	Low	34.17	$\chi^{2}=$ 314.3
		Middle	29.58	
		High	49.30	
	Having books	Little	22.45	$\chi^{2}=$ 1401.32
		Less	27.16	
		General	40.08	
		More	59.47	
		Many	70.59	
	Having computers and networks	None	24.65	$\chi^{2}=$ 1121.21
		One of them	46.48	
		Both	56.20	
	Parents’ academic assistance	Yes	48.38	$\chi^{2}=$ 195.38
		No	33.39	
School level	School ranking	Middle and lower	33.19	$\chi^{2}=$ 436.62
		Upper	43.38	
		Great	61.22	
	School type	Public school	45.82	$\chi^{2}=$ 39.19
		Private school	33.97	

Due to space limitations, some variables are not included. In addition, Parents’ Education Level’, ‘Self-Education Expectation’, and Parents’ Education Expectation’ are defined as low in “lack of education,” “elementary school,” and “junior” while “technical art school/technical school,” “vocational high school,” and “high school” are defined as “Middle,” and “College,” “Bachelor,” and “Graduate and Above” are defined as “Highly Educated.” All *p* values corresponding to the Chi-Square Test in the table are less than 0.

Family economic levels and parents’ class levels both increased the possibility of participating in shadow education. The participation rates of rich families and the advantaged class were higher than that of poor families and lower-class families. Parents’ educational expectations and students’ self-education expectations were consistent, indicating that educational expectation may be an important factor affecting the heterogeneity of shadow education. The proxy variables of family cultural capital showed that the more books and internet access available in the household, the more importance parents gave to their children’s studies, potentially increasing participation in shadow education and having a positive effect on its acquisition.

At the school level, the proportion of shadow education in public schools was significantly higher than that in private schools, and it has been increasing with the rise of school rankings. In Table 2, the heterogeneous factors at the three levels all passed the Chi-Square independence test, indicating a connection between the differences of micro-heterogeneous factors and the acquisition of shadow education, and that individual heterogeneity may be an important factor affecting individuals’ participation in shadow education. However, the strength of rejection to the null hypothesis differed, the preliminary findings further clarified that family factors may be the key type of heterogeneity affecting the acquisition of shadow education.

**Figure 1:**
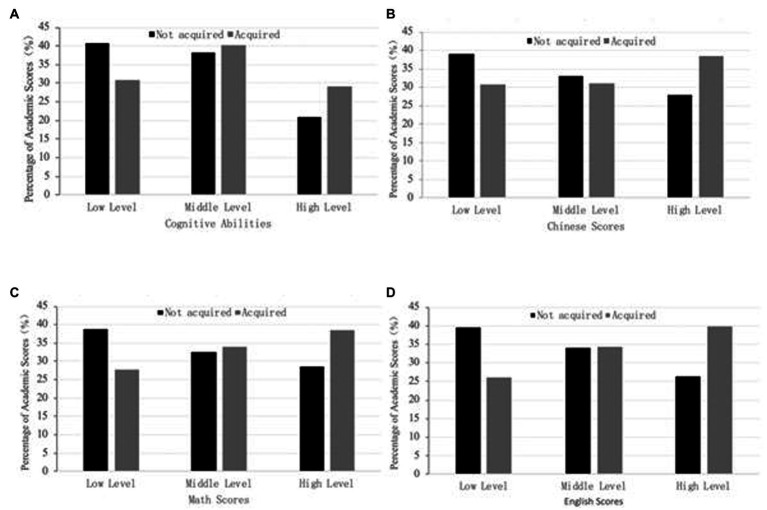
Shadow education acquisition and the percentage of academic performance. **(A)** Shadow education acquisition and cognitive abilities. **(B)** Shadow education acquisition and Chinese scores. **(C)** Shadow education acquisition and math scores. **(D)** Shadow education acquisition and English scores.

Figures 1A–D shows the initial relationship between shadow education and student achievement levels. We divided achievement into three levels, low, middle, and high, and constructed four different types of educational results. The Chi-Square independence tests in four aspects were all passed at a significance level of 1%.

The proportion of students with high cognitive ability increased significantly, while the proportion of students with low cognitive ability decreased. The horizontal distribution of cognitive ability was of the “U” type; therefore, shadow education brings cognitive change, as reflected in the high levels of cognitive ability. Educational results and cognitive ability are different: Low-level students accounted for the largest proportion in the group that did not participate in shadow education, while the high-level students accounted for the largest proportion in the group that participated. This indicates that the acquisition of shadow education may have increased students’ achievement in all major subjects, focusing on high-level students. Shadow education can improve the performance of students at the middle- and high-levels, among them, the improvement of Chinese scores is only higher among the groups of students who are good at learning. In other words, students participating in shadow education only target minority groups in terms of improving their language scores. The math scores and English scores are slightly similar. The acquisition of shadow education will increase the proportion of students at the middle and high levels. At the same time, the reduction of shadow education on the proportion of low-level math scores is significantly smaller than the effect on English. Improvements to the former will reach a wider group than the latter. This is because shadow education may improve mathematics performance more timely and efficiently, while it may have a “rewarding” effect on English performance.

### Shadow education: Test of the mediation effect

Through the aforementioned independence test, we can roughly draw the possible correlation among the heterogeneous factors, shadow education, and educational results. Xue (2018) constructed a theoretical model based on the heterogeneous factors in family capital, pointing out that it can affect both children’s educational results at school and influence their shadow education opportunities. Thus, it is necessary to test which factors will be affected by the mediation effect, while the mediation effect of shadow education needs to be tested at different levels.

#### Model specification

Mediation effect analysis is a mechanism judgment method that is widely used in many fields. Since the mediation variable in this study was a binary variable with values of 0–1, we therefore used Logit regression and the maximum likelihood method (ML) for the preceding part of the mediation, while OLS was used for the latter part.


(1) $$Score^{j}=\alpha +\phi \mathrm{Factors}+\varepsilon$$

(2) $$\ln\left(\frac{P_{shadow}}{1-P_{shadow}}\right)=\beta +\psi \mathrm{Factors}+\mu$$

(3) $$Score^{j}=\gamma +\phi^{’}\mathrm{Factors}+\Omega \mathrm{Shadow}+\tau$$

**Figure 2:**
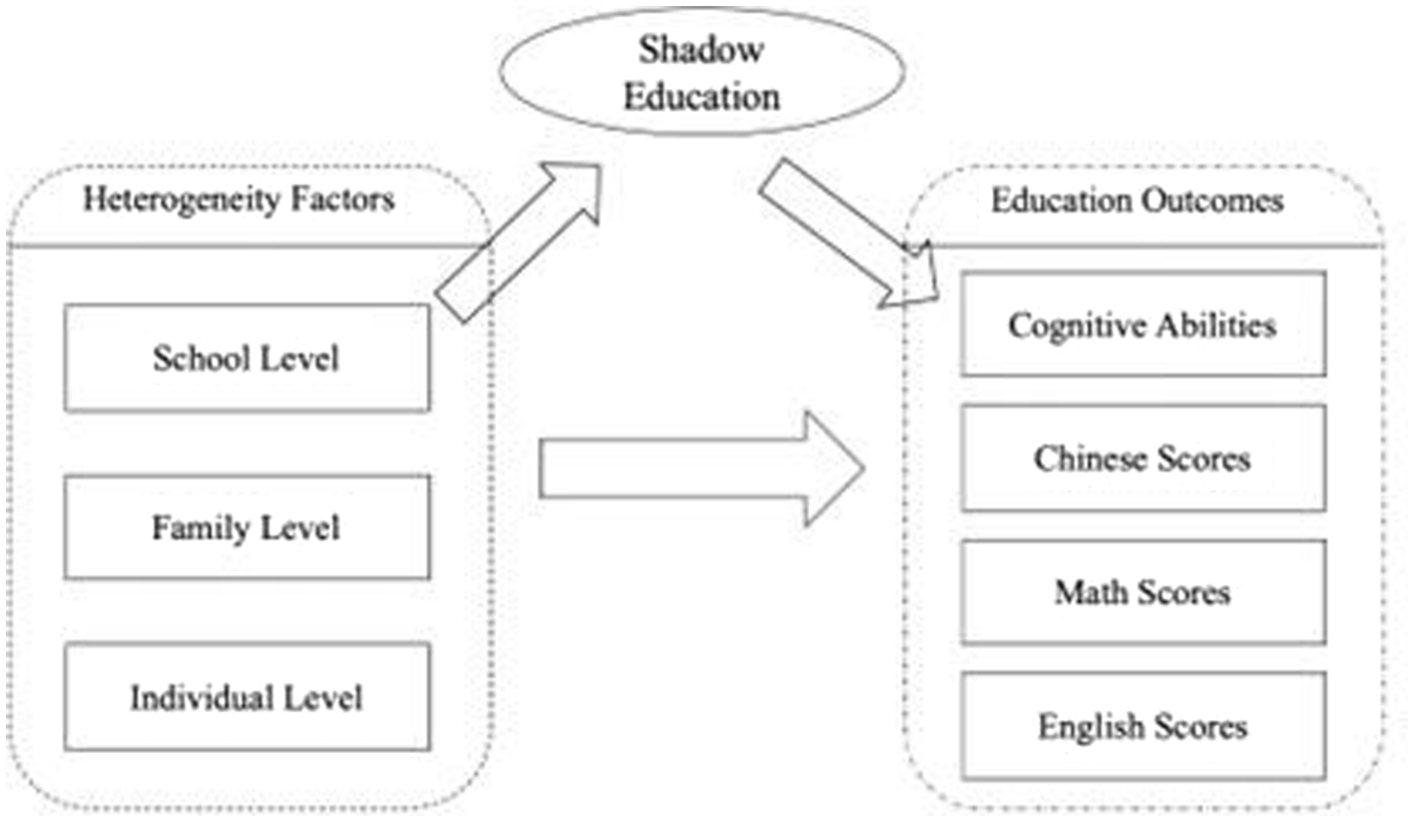
Shadow education mediation effect.

$Score^{j}$ represents students’ educational results and contains four categories. Factors represent heterogeneous factors at the individual, family, and school levels. Shadow represents shadow education acquisition, and $P_{shadow}$ indicates the student’s probability of shadow education acquisition. ε , μ, and τ are the errors of the three models, respectively. Figure 2 shows a more intuitive demonstration of the mediation effect.

Baron and Kenny (1986) causal regression method is the traditional test method of mediation effect. In other words, the significance of the coefficients in the above three formulas was tested sequentially; however, many studies have questioned this method (Zhao et al., 2010; Iacobucci, 2012). Some scholars have proposed that the product of coefficients of equations (2) and (3) should be tested for the mediation effect. Sobel (1982) gave the test statistics under the condition that ψΩ has a normal distribution and pointed out that its test effectiveness was obviously superior to the above causal regression test method. However, the normality assumption is too strict to be easily satisfied in practical application, thus increasing the probability of making the first type of error. To further ease the assumption of the Sobel test, Preacher and Hayes (2004) proposed the mainstream Bootstrap method for the mediation effect test which is called the non-parametric percentile Bootstrap method. The Sobel test and non-parametric percentile Bootstrap method results are reported simultaneously. Since the percentile information interval for bias correction is asymmetric and better reflects the sampling distribution of conditional indirect effects, we further report the non-parametric percentile Bootstrap method for bias correction (Fritz et al., 2012; Hayes and Scharkow, 2013), estimating the resampling number at 1,000. A generalized structural equation model (GSEM) which can deal with explicit variables and latent variables simultaneously, and analyze the relationship among multiple independent, dependent, and mediation variables simultaneously (Fang et al., 2014) was used to evaluate the mediation effect of shadow education. To ensure that the statistical test of the above three equations was robust, we clustered the errors into the class level.

### The mediation effect test of shadow education

Table 3 shows the mediation effect of shadow education on students’ cognitive ability from the heterogeneous factors perspective.

**Table 3:** Mediation effect of shadow education (cognitive ability).

Variable name	Point estimation	Interval estimation
	Mediation effect	Sobel test		Bootstrap method		Deviation correction by bootstrap method	
School ranking	−0.0376	−0.0638	−0.0113	−0.0657	−0.0149	−0.0668	−0.0158
School type	−0.0609	−0.1199	0.002	−0.1293	−0.0111	−0.1471	−0.0162
School size	−0.0609	−0.1179	−0.004	−0.1239	−0.0115	−0.1248	−0.0137
Class size	−0.0034	−0.0071	0.0004	−0.0073	−0.0001	−0.0076	−0.0001
Teacher numbers	−0.0006	−0.0011	−0.0001	−0.0012	−0.0001	−0.0012	−0.0001
Family economic status	−0.0418	−0.0734	−0.0103	−0.0795	−0.0139	−0.0805	−0.0145
Parents’ occupation	−0.0865	−0.1297	−0.0434	−0.1334	−0.0457	−0.1333	−0.0451
Parents’ education	−0.027	−0.041	−0.013	−0.0417	−0.0137	−0.0416	−0.0137
Parents’ political status	−0.0391	−0.0789	0.0007	−0.0823	−0.0055	−0.096	−0.0091
Parents’ education expectation	−0.018	−0.03	−0.0061	−0.0317	−0.0081	−0.0321	−0.0082
Having books	−0.0674	−0.0999	−0.0348	−0.1013	−0.0355	−0.1015	−0.0357
Having computers and networks	−0.0619	−0.0946	−0.0292	−0.0954	−0.0313	−0.0994	−0.0327
Parents’ academic assistance	−0.0779	−0.1228	−0.0329	−0.1281	−0.0382	−0.1324	−0.041
Gender	0.0547	0.02	0.0894	0.0247	0.0957	0.0255	0.0965
Grade	0.0391	0.0108	0.0674	0.0143	0.071	0.0165	0.075
Ethnicity	0.0472	−0.0039	0.0983	0.0011	0.1022	0.0001	0.1018
Hukou	0.0756	0.0335	0.1178	0.0348	0.1187	0.0386	0.1223
one child	−0.074	−0.1167	−0.0314	−0.1165	−0.0338	−0.1188	−0.0353
Performance ranking	0.0118	−0.0004	0.0241	0.001	0.0257	0.0018	0.0286
Self-education expectation	−0.0055	−0.0142	0.0032	−0.015	0.0021	−0.0163	0.0017

Overall, the Sobel test rejected the existence of the shadow education mediation effect in some variables; however, a more accurate non-parametric percentile Bootstrap method and deviation-corrected non-parametric percentile Bootstrap method reached different conclusions. Although individual-level self-education expectation was not mediated by shadow education, all the other heterogeneous factors at the three levels had certain mediation effects. From the test results and point estimates of indirect effects, the confidence interval was close to zero; however, the absolute amount of indirect effects was very small. Therefore, we have reason to believe that although shadow education had a mediation effect on the heterogeneous factors’ influence on students’ cognitive ability, and it passed the statistical test, the mediation effect can be ignored in the economic sense. Table 4 shows the mediation role of shadow education on students’ Chinese scores from the heterogeneous factors perspective. It is obvious that the null hypothesis ψΩ=0 could not be rejected under the three different testing methods, indicating that the school-, family-, and individual-level heterogeneous factors were not mediated by shadow education: It had no significant mediation effect on Chinese scores, and the educational results did not change due to the differences in the acquisition of shadow education brought about by heterogeneity. Therefore, there was no inequality of educational results in this respect.

**Table 4:** Mediation effect of shadow education (Chinese scores).

Variable name	Point estimation	Interval estimation
	Mediation effect	Sobel test		Bootstrap method		Deviation correction by bootstrap method	
School ranking	0.0600	−0.0411	0.1611	−0.0344	0.1758	−0.0211	0.1922
School type	0.0973	−0.0846	0.2792	−0.0517	0.3096	−0.0400	0.3262
School size	0.0973	−0.0742	0.2688	−0.0519	0.2954	−0.0244	0.3388
Class size	0.0054	−0.0064	0.0171	−0.0038	0.0190	−0.0021	0.0221
Teacher number	0.0009	−0.0008	0.0026	−0.0007	0.0029	−0.0003	0.0034
Family economic status	0.0668	−0.0444	0.1779	−0.0398	0.1902	−0.0283	0.1999
Parents’ occupation	0.1382	−0.0746	0.3509	−0.0794	0.3499	−0.0838	0.3444
Parents’ education	0.0431	−0.0228	0.1090	−0.0185	0.1120	−0.0225	0.1073
Parents’ political status	0.0625	−0.0539	0.1788	−0.0294	0.1938	−0.0148	0.2121
Parents’ education expectation	0.0288	−0.0192	0.0768	−0.0153	0.0804	−0.0134	0.0834
Having books	0.1075	−0.0558	0.2708	−0.0534	0.2709	−0.0546	0.2705
Having computers and networks	0.0988	−0.0580	0.2556	−0.0466	0.2619	−0.0463	0.2646
Parents’ academic assistance	0.1243	−0.0752	0.3238	−0.0621	0.3350	−0.0640	0.3330
Gender	−0.0874	−0.2294	0.0547	−0.2373	0.0539	−0.2362	0.0541
Grade	−0.0624	−0.1593	0.0344	−0.1653	0.0254	−0.1834	0.0170
Ethnicity	−0.0753	−0.2210	0.0704	−0.2521	0.0393	−0.2854	0.0195
Hukou	−0.1207	−0.3134	0.0720	−0.3345	0.0713	−0.3442	0.0563
One child	0.1181	−0.0694	0.3057	−0.0538	0.3169	−0.0316	0.3373
Performance ranking	−0.0189	−0.0550	0.0172	−0.0610	0.0089	−0.0684	0.0043
Self-education expectation	0.0088	−0.0124	0.0299	−0.0063	0.0374	−0.0038	0.0413

Table 5 shows the mediation effect of shadow education on students’ math scores from the heterogeneous factors perspective. The main mediation effects of shadow education at the school level were found to be school ranking, type, and size, while the parental occupation at the family level had the largest mediation effect, with a total indirect effect of 0.4357. This indicated that the higher the occupation level of the parents, the higher the degree of participation in shadow education. Additionally, family economic status at the family level – books representing cultural capital, computers and internet access, and parental academic assistance – all had significant effects on the inequality of educational results in math scores by influencing shadow education acquisition. In contrast, shadow education did not have any obvious mediation effects on parents’ educational levels, political status, or educational expectations. Hukou, gender, and one-child status were the main mediated factors.

**Table 5:** Mediation effect of shadow education (math scores).

Variable name	Point estimation	Interval estimation
	Mediation effect	Sobel test		Bootstrap method		Deviation correction by bootstrap method	
School ranking	0.1891	0.0267	0.3515	0.0454	0.3777	0.0569	0.3897
School type	0.3068	−0.0319	0.6454	0.0386	0.7128	0.0697	0.8246
School size	0.3068	−0.0335	0.6471	0.0298	0.7209	0.0690	0.7845
Class size	0.0170	−0.0053	0.0392	−0.0009	0.0425	0.0004	0.0454
Teacher number	0.0029	−0.0003	0.0062	0.0003	0.0064	0.0005	0.0069
Family economic status	0.2106	0.0140	0.4072	0.0474	0.4309	0.0594	0.4662
Parents’ occupation	0.4357	0.0920	0.7794	0.0847	0.7936	0.0995	0.8151
Parents’ education	0.1359	0.0321	0.2397	0.0355	0.2458	0.0385	0.2509
Parents’ political status	0.1970	−0.0319	0.4259	0.0094	0.4594	0.0274	0.5195
Parents’ education expectation	0.0908	0.0133	0.1684	0.0224	0.1817	0.0256	0.1900
Having books	0.3391	0.0987	0.5794	0.0830	0.5755	0.1086	0.6106
Having computers and networks	0.3116	0.0774	0.5459	0.0894	0.5616	0.0838	0.5492
Parents’ academic assistance	0.3920	0.0742	0.7098	0.1191	0.7557	0.1382	0.7629
Gender	−0.2755	−0.4953	−0.0558	−0.5112	−0.0737	−0.5337	−0.0904
Grade	−0.1969	−0.3797	−0.0141	−0.4068	−0.0404	−0.4782	−0.0553
Ethnicity	−0.2376	−0.5395	0.0644	−0.5796	−0.0022	−0.6141	−0.0153
Hukou	−0.3807	−0.6866	−0.0748	−0.7193	−0.1097	−0.7230	−0.1157
One Child	0.3725	0.0769	0.6680	0.1026	0.6810	0.1068	0.7024
Performance ranking	−0.0596	−0.1279	0.0087	−0.1368	−0.0078	−0.1529	−0.0126
Self-Education expectation	0.0277	−0.0184	0.0738	−0.0122	0.0830	−0.0066	0.0909

Table 6 shows the corresponding results for English scores, which were similar to those of cognitive ability, except that self-education expectations at the individual level were not significant. All the heterogeneity factors in other levels passed the test. As Table 6 shows, the school- and family-level tests were consistent with those based on math scores. Among them, school ranking, type, and size; parents’ occupation; family economic status; books and internet access; and parents’ academic assistance in family economic level had stronger mediation effects. However, compared with the results in Table 5, the mediation effect strength of the above factors decreased. The heterogeneous factors at the individual level are significantly enhanced by mediation effects.

**Table 6:** Mediation effect of shadow education (English scores).

Variable name	Point estimation	Interval estimation
	Mediation effect	Sobel test		Bootstrap method		Deviation correction by bootstrap method	
School ranking	0.3036	0.1011	0.5062	0.1209	0.5246	0.1335	0.5556
School type	0.4926	0.0474	0.9378	0.0923	0.9594	0.1164	1.0169
School size	0.4926	0.0412	0.9441	0.0893	1.0288	0.1056	1.0484
Class size	0.0272	−0.0036	0.0580	0.0009	0.0603	0.0021	0.0638
Teacher number	0.0047	0.0005	0.0089	0.0007	0.0091	0.0009	0.0095
Family economic status	0.3381	0.1032	0.5731	0.1307	0.5911	0.1529	0.6332
Parents’ occupation	0.6996	0.3823	1.0169	0.3831	1.0268	0.4169	1.0748
Parents’ education	0.2182	0.1127	0.3238	0.1239	0.3264	0.1239	0.3261
Parents’ political status	0.3164	−0.0146	0.6473	0.0310	0.6983	0.0527	0.7543
Parents’ education expectation	0.1459	0.0550	0.2368	0.0694	0.2494	0.0714	0.2542
Having books	0.5445	0.2891	0.7999	0.3070	0.8168	0.3389	0.8752
Having computers and networks	0.5004	0.2622	0.7386	0.2778	0.7458	0.2901	0.7585
Parents’ academic assistance	0.6294	0.2882	0.9707	0.3378	1.0098	0.3445	1.0225
Gender	−0.4424	−0.7021	−0.1827	−0.7360	−0.2177	−0.7603	−0.2314
Grade	−0.3162	−0.5180	−0.1143	−0.5272	−0.1324	−0.5781	−0.1501
Ethnicity	−0.3815	−0.7736	0.0107	−0.8347	−0.0289	−0.8501	−0.0394
Hukou	−0.6112	−0.9277	−0.2948	−0.9424	−0.3206	−0.9699	−0.3331
One child	0.5981	0.2821	0.9140	0.3168	0.9591	0.3256	0.9765
Performance ranking	−0.0957	−0.1918	0.0003	−0.1996	−0.0121	−0.2065	−0.0155
Self-education expectation	0.0445	−0.0235	0.1124	−0.0186	0.1152	−0.0187	0.1151

Testing the specific heterogeneous factors of shadow education as mediation showed that shadow education had no significant mediation effect on cognitive ability and Chinese scores but some effect on math and English scores. The results of testing math scores indicated that family-level heterogeneous differences were the key factor affecting the probability of acquiring shadow education and, thus, leading to inequality in students’ math scores. Family factors (compared with other levels) were also an important source of inequality in English scores, with a relatively increased mediation degree when compared with math scores. The role of heterogeneous factors at the individual level was also highlighted here.

### Education finance: Examining moderation effects

Through the above investigation, we concluded that shadow education plays a part in mediating the effect of heterogeneous factors on educational results and has a significant effect on math and English scores. Above all, school ranking, type, and size; parents’ occupation; family economic status; books and internet access; parents’ academic assistance; hukou type; gender; and one-child family status were the key factors being mediated. We then further investigated whether macro-national education finance has a negative moderation effect on the educational results inequality brought about by shadow education.

#### Model specification

**Figure 3:**
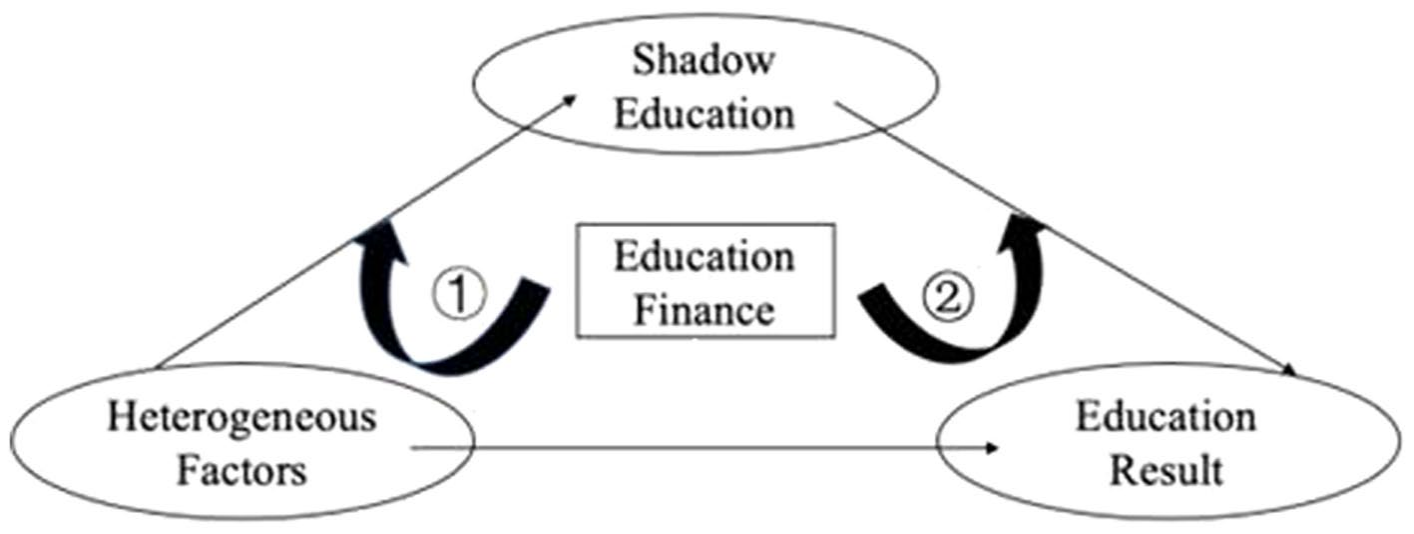
The moderation path of education finance.

Based on the above constructed mediation effect model, three mediation effect models including moderation were established for estimation. We investigated whether education finance had a moderation effect in the process of shadow education mediation and clarified the specific moderation path and direction. Figure 3 shows the specific moderation path of education finance in the process of shadow education mediation.

For a certain heterogeneous factor, ① and ② respectively represent the preceding and latter paths of moderation effects. If education finance produced moderation in both paths, it was considered to have a moderation effect on the whole path. The specific test model was set as follows:

(1) Whole Path Moderation (① and ②)

$$\{\begin{array}{c} \ln\left(\frac{P_{shadow}}{1-P_{shadow}}\right)=\alpha_{0}+\alpha_{1}Factors+\alpha_{2}R+\alpha_{3}Factors*R\\ \begin{array}{l} Score^{j}=\beta_{0}+\beta_{1}Shadow+\beta_{2}Factors+\beta_{3}R\\ +\beta_{4}Factors*R+\beta_{5}Shadow*R \end{array}\\ Conditionaleffect=\left(\alpha_{1}+\alpha_{3}R\right)\left(\beta_{1}+\beta_{5}R\right) \end{array}$$

(2) Preceding Moderation (Only ①)

$$\{\begin{array}{c} \ln\left(\frac{P_{shadow}}{1-P_{shadow}}\right)=\alpha_{0}+\alpha_{1}Factors+\alpha_{2}R+\alpha_{3}Factors*R\\ Score^{j}=\beta_{0}+\beta_{1}Shadow+\beta_{2}Factors+\beta_{3}R+\beta_{4}Factors*R\\ Conditionaleffect=\left(\alpha_{1}+\alpha_{3}R\right)\beta_{1} \end{array}$$

(3) Latter Moderation (Only ②)

$$\{\begin{array}{c} \ln\left(\frac{P_{shadow}}{1-P_{shadow}}\right)=\alpha_{0}+\alpha_{1}Factors\\ Score^{j}=\beta_{0}+\beta_{1}Shadow+\beta_{2}Factors+\beta_{3}R+\beta_{5}Shadow*R\\ Conditionaleffect=\alpha_{1}\left(\beta_{1}+\beta_{5}R\right) \end{array}$$

Most variables have the same meaning as above, R represents financial access, and Conditionaleffect is the conditional mediation effect. The mediation effect containing the moderation can also be estimated by the structural equation model (Bolin, 2014). Wen and Ye (2014) gave a detailed test procedure, and, according to the test results, the deviation correction non-parametric percentile Bootstrap method was used for judgment.

### Testing the moderation effect of education finance

Judging whether the confidence interval of $\alpha_{3}\beta_{5},\alpha_{3}\beta_{1}$ , and $\;\alpha_{1}\beta_{5}$ in the corresponding model contained zero was used to test whether education finance had a full path moderation effect, and the specific moderation path that existed. Table 7 shows the test results represented by math and English scores. Among the three levels, the influence of heterogeneous factors on educational results through shadow education was moderated by national education finance. Moreover, the mediation heterogeneous factors had obvious differences in the distribution of education finance moderation. In the educational results represented by math scores, all factors of education finance moderation acted on the preceding path, while in terms of English scores, the moderation effect of education finance was more abundant. In the investigation of certain heterogeneous factors, education finance also demonstrated the moderation effect of the latter path.

**Table 7:** Path test of the moderation effect of national education finance.

Heterogeneous levels	Heterogeneous factors	Math scores		English scores	
		Preceding	Latter	Preceding	Latter
School level	School ranking	√	✕	√	✕
	School type	√	✕	√	✕
	School size	✕	✕	✕	✕
Family level	Family economic status	√	✕	✕	√
	Parents’ occupation	√	✕	√	✕
	Having books	√	✕	√	✕
	Having computers and networks	✕	✕	✕	√
	Parents’ academic assistance	✕	✕	✕	√
Individual level	Gender	✕	✕	✕	√
	Hukou	✕	✕	✕	√
	One child	✕	✕	✕	√

According to the corresponding test model and the 95% confidence interval obtained by the non-parametric percentile Bootstrap method of deviation correction, if the interval contains zero, then the moderation effect does not exist on the path, and vice versa.

Specifically, under the educational results represented by math scores, school-level factors, computer network ownership, and parental academic assistance at the family and individual levels were not moderated by education finance in any of the shadow education mediation paths. School ranking and type, family economic status, parents’ occupation, and having books all had preceding moderation effects on education finance. However, with the educational results represented by English scores, the mediation-moderation effect of education finance to most of the heterogeneous factors in shadow education was generated in the latter path, while school size was also not affected by the moderation effect. Unlike the results of the math test, this test’s results showed the latter path’s moderation to a greater extent and highlighted the moderation effect of the heterogeneous factors at the individual level. It can be concluded that the finance moderation effect of the heterogeneous factors affecting the inequality of educational results through shadow education mediation has different dimensions and paths.

The above test shows which factors were moderated by the education finance and specific moderation path while being mediated by shadow education. We further investigated the adjustment direction and effect size of education finance by obtaining the specific values at the mean and the next standard deviation of education finance, substituting the corresponding expressions for the conditional mediation effect, and achieving the corresponding results by the magnitude and direction of values (Preacher et al., 2007).

Table 8 shows the effect and direction of education finance moderation. Whether regarding math or English scores, the moderation degree of education finance on different levels of heterogeneous factors was significantly different, and the effect on family-level factors was relatively large. Specifically, when using math scores for the estimation of the educational results, with the increase of financial input in education, the mediation effect of school level, parent occupation, and having books (representing cultural capital) decreased. This shows that education finance to some extent reduces the inequality of educational results caused by these three heterogeneous factors. Education finance reduced the education investment of the low economic status and occupational class and the inequality of educational results, and the effect of shadow education mediation also appeared to be reduced. Table 8 also shows that the shadow education mediation effect of school type increases with education input; in other words, public school students are more likely to increase their demand for shadow education when their external investment increases, and this micro-individual behavior affects the equality of educational results.

**Table 8:** The moderation effect of national education finance.

Educational outcomes	Heterogeneous factors	Mean−1SD	Mean	Mean + 1sd	Moderation direction	Moderation paths
Math scores	School ranking	0.2585**	0.1714**	0.0843	−	Preceding
	School type	0.1494	1.0351**	1.9208**	+	Preceding
	Family economic status	0.2553***	0.1978***	0.1402*	−	Preceding
	Parents’ occupation	0.4952***	0.4078***	0.3204**	−	Preceding
	Having books	0.3627**	0.3074**	0.2522**	−	Preceding
English scores	School ranking	0.4561***	0.3024***	0.1487	−	Preceding
	School type	0.2479	1.7179***	3.1879***	+	Preceding
	Family economic status	0.5385***	0.3296***	0.1207*	−	Latter
	Parents’ occupation	0.8306***	0.684***	0.5374***	−	Preceding
	Having books	0.6132***	0.5198***	0.4263***	−	Preceding
	Having computers and networks	0.1787	0.4878***	0.7969***	+	Latter
	Parents’ academic assistance	0.2248	0.6136***	1.0023***	+	Latter
	Gender	−0.158	−0.4312***	−0.7045***	+	Latter
	Hukou	−0.2183	−0.5958	−0.9734	+	Latter
	One child	0.2136	0.5829***	0.9524***	+	Latter

‘*’, ‘**’, ‘***’ represent significant at the 1%, 5%, and 10% significance levels, respectively.

Compared with the estimated results of math scores, the moderation effect of education finance on English scores was more obvious. However, the direction of the moderation effect did not change, showing that the moderation effect of education finance does not change with the different types of educational results. At the same time, the results of the other two proxy variables of family cultural capital showed that education finance positively moderates the probability that families with computer networks and higher academic guidance participate in shadow education, thus increasing the difference between students’ English scores. The differences between the three heterogeneous factors at the individual level affected the mediation process of educational results, which was also moderated by education finance. Education finance may enlarge the shadow education mediation effect of the gender and urban–rural attributes and further affect the equality of educational results. However, this result was affected by the sample structure; thus, it does not have sufficient policy value but proves that education finance plays an important role therein. Additionally, students from one-child families with the same education finance subsidy have a more obvious motivation to change their results through shadow education.

## Conclusion and discussion

From the perspective of shadow education mediation, we used China’s CEPS database to empirically study the effects of micro-heterogeneity factors on the equality of educational results and whether education finance reduces the potential unequal educational results caused by shadow education. From the preliminary investigation of the relationship among heterogeneous factors, shadow education acquisition, and educational results, we drew two conclusions. First, there is a connection between the differences in micro-heterogeneity factors and the acquisition of shadow education, and individual heterogeneity may be an important factor affecting individuals’ participation in shadow education. We observed that family factors may be the key type of heterogeneity affecting the acquisition of shadow education, which also verifies the findings of Xue (2015) and Xue and Li (2016). Meanwhile, there is a strong correlation between shadow education and educational results, although performance varies in different courses. We conclude that the distribution of cognitive ability presents an ‘inverted U’ type, while shadow education gains may have increased student achievement in all three other subjects. Among these, shadow education has no extensive effect on the improvement of Chinese scores, while it has the opposite effect on math scores and may play a role in cultivating excellent students in English courses.

It was also found that shadow education has no mediation effect on the educational results represented by cognitive ability and Chinese scores. The former effect was very small, while the latter was not significant at all. The mediation effect of shadow education mainly exists in the educational results represented by math and English scores. The differences in individual heterogeneity have an adverse effect on the equality of educational results by allowing additional opportunities for participation in shadow education: School ranking, type, and school size; parents’ occupation; family economic status; having books, computers, and internet access; and parents’ academic assistance have strong mediated effects. In addition, the mediation process of heterogeneous factors at the individual level is relatively significant in the examination of English scores.

Finally, this study examines whether education finance can reduce the inequality of educational outcomes brought about by shadow education. By constructing a mediating effect model that includes moderating effects, on the one hand, we test whether the moderating effect of education finance exists, and on the other hand, it is also clear that Specific adjustment path and adjustment direction. We draw the following conclusions: (1) Heterogeneous factors are mediated by shadow education, and the fiscal moderating effect that affects inequality of educational outcomes has different dimensions and paths. Specifically, regardless of the type of educational outcome, education finance has an antecedent moderating effect on school ranking and type, parental occupation level, and family book ownership. The adjustment paths of household economic level are different. In addition, individual-level factors are only affected by the adjustment of education finance in the test of educational results represented by English achievement, and they are all adjusted in the latter stage. (2) Educational finance has significantly different adjustment degrees and directions on various heterogeneity factors at different levels, and it has a relatively large adjustment effect on the differences in family-level factors through shadow education, thereby forming inequality in educational outcomes. Specifically, education finance reduces the inequitable educational outcomes caused by differences in economic level, parental occupation level, and family book collection, while family students in public schools are more likely to increase their demand for shadow education when exogenous input increases. Micro-individual behavior affects the realization of fairness in educational outcomes. At the same time, the moderating effect is more obvious under the condition that English achievement is the object of investigation, but the moderation direction does not change, indicating that the moderating effect of education finance in the mediating process is relatively stable. In addition, the effect on the individual level has no effect. Sufficient policy reference value.

The above conclusions give us certain policy implications. First of all, the government needs to pay more attention to the important role of shadow education in the realization of educational equity, especially to clarify its mediating role in the impact of micro-heterogeneous differences on the inequality of educational outcomes, and to carry out special projects on shadow education. The rectification work should reduce the adverse effects of shadow education, take the fair access to shadow education as the main development direction in the future, and maximize the advantages of shadow education in providing high-quality educational resources for relatively disadvantaged groups. Secondly, the main conclusion of this paper is that the national education financial investment can alleviate the inequality of educational outcomes caused by shadow education intermediaries, especially the inequity in education caused by the heterogeneity of family capital. Therefore, education finance should be further moderately biased towards disadvantaged family capital groups, which may reduce the adverse impact of non-standardized shadow education on the fairness of educational outcomes to a certain extent.

## Data availability statement

Publicly available datasets were analyzed in this study. This data can be found at: http://ceps.ruc.edu.cn/.

## Author contributions

YM: conceptualization and writing-original draft. WJ: software and revising original draft. JW and XW: methodology and revising original draft. YZ: software, methodology, writing-original draft, and project administration. ZY: software, methodology, and writing-original draft. All authors contributed to the article and approved the submitted version.

## Funding

This research was financially supported by the Postgraduate Education Innovation Plan of Anhui University of Finance and Economics (project number: cxjhjyyb2214).

## Conflict of interest

The authors declare that the research was conducted in the absence of any commercial or financial relationships that could be construed as a potential conflict of interest.

## Publisher’s note

All claims expressed in this article are solely those of the authors and do not necessarily represent those of their affiliated organizations, or those of the publisher, the editors and the reviewers. Any product that may be evaluated in this article, or claim that may be made by its manufacturer, is not guaranteed or endorsed by the publisher.
